# Understanding Esophageal Cancer: The Challenges and Opportunities for the Next Decade

**DOI:** 10.3389/fonc.2020.01727

**Published:** 2020-09-10

**Authors:** Jianjun Yang, Xiguang Liu, Sai Cao, Xiaoying Dong, Shuan Rao, Kaican Cai

**Affiliations:** Department of Thoracic Surgery, Nanfang Hospital, Southern Medical University, Guangzhou, China

**Keywords:** epidemiology, esophageal cancer, risk factors, genetics, clinical management, immunotherapy

## Abstract

Esophageal cancer (EC) is the seventh most common cancer worldwide with over 570,000 new cases annually. In China, the incidence of EC is particularly high where approximately 90% of cases are defined as esophageal squamous cell carcinoma (ESCC). Although various risk factors have been identified, the knowledge of genetic drivers for ESCC is still limited due to high mutational loading of the cancer and lack of appropriate EC models, resulting in inadequate treatment choices for EC patients. Currently, surgery, chemotherapy, radiation, and limited targeted therapy options can only bring dismal survival advantages; thus, the prognosis for ESCC is very poor. However, cancer immunotherapy has unleashed a new era of cancer treatment with extraordinary therapeutic benefits for cancer patients, including EC patients. This review discusses the latest understanding of the risk factors and clinical rational for EC treatment and provides accumulated information, which describes the ongoing development of immunotherapy for EC with a specific emphasis on ESCC, the most prevalent EC subtype in the Chinese population.

## Introduction

Esophageal cancer (EC) is ranked as the seventh most common cancer worldwide with over 570,000 new cases in 2018 (1). The pathology of EC is relatively less understood compared to many other cancers, and it usually shows extremely aggressive clinical features when diagnosed; thus, it is known that EC is the sixth leading cause of malignancy-related death with a 5-year survival ratio of 15–20% (1).

There are two major subtypes of EC, esophageal squamous cell carcinoma (ESCC) and esophageal adenocarcinoma (EAC), which usually occur in either proximal ESCC or distal esophagus EAC, respectively (2). Although ESCC is the predominant pathological type of EC, the incidence of ESCC and EAC can be very different among different countries and regions (3). For instance, ESCC patients account for 90% of cases in China, Japan, and southeast Africa countries (4–6); however, EAC is more prevalent in the United States, Australia, and Western European countries (7–9). Recently, accumulated evidence has suggested that ESCC and EAC are actually two different diseases (10, 11), as they have quite different risk factors and genetic profiles; however, EAC is more comparable to gastroesophageal junction carcinomas (GEJCs) or gastric cancer (GC). In this review, we will particularly discuss ESCC, the subtype diagnosed for more than 250,000 patients every year in China.

## The Epidemiology of Esophageal Cancer in China

Esophageal cancer is a significant public health burden in China, although some recent studies have indicated that the incidence of EC is decreasing in the last few decades. In 2012, China contributed nearly half of the global new EC patients. Briefly, EC was the fifth most frequently diagnosed cancer and the fourth leading cause of cancer-related death in China, with estimated 286,700 new and 211,000 death cases (12). Another study indicated that there were 276,900 newly diagnosed and 206,500 death EC cases in 2013; the incidence was 28.15/10^5^ and 12.15/10^5^ in male and female, respectively (13), which decreased to 26.46/10^5^ and 10.85/10^5^ in 2014 (14). Furthermore, in 2015, it was estimated that the occurrence of EC was 17.87/10^5^ and a rough mortality of 13.68/10^5^ in the Chinese population (15). Indeed, the statistics from the National Central Cancer Registry of China (NCCRC) also showed a decreasing trend of EC incidence and mortality in both male and female populations from 2000 to 2013 (13). Of note, the incidence and mortality of EC were quite different among different regions in China. Provinces near the Taihang Mountains, such as Hebei, Henan, and Chongqing had a relatively higher EC incidence, whereas in Xinjiang, Jiangsu, Shanxi, Gansu, and Anhui, the EC reported cases were much less (16).

ESCC is the predominant histological subtype of EC in China, accounting for 88.84% of all EC cases in the Chinese population (17). Although increasing survival of EC patients were observed in both population-based and hospital-based studies (18, 19), the prognosis for ESCC/EC in China is relatively poor when compared to other cancers, as most studies reported that EC patients had a 5-year survival rate between 15 and 25% (20, 21). In a population-based study including 1,033 ESCC patients who received surgery, patients in stages IA, IB, IIA, IIB, IIIA, IIIB, IIIC, and IV had a 5-year survival rate of 84.9, 70.9, 56.2, 43.3, 37.9, 23.3, 12.9, and 3.4%, respectively (22). Similar findings were reported in another Chinese population-based study (23). Importantly, the survival for those ESCC patients with distant organ metastasis at the first diagnosis is particularly poor, as a retrospective study indicated that these patients had a median survival of only 6 months with 1- or 2-year survival rates of 21.1 or 11.8% (24), respectively. Furthermore, hospital-based studies also suggested that the 5-year survival rate of EC patients was <20% (19, 25).

## Risk Factors of EC

**Figure 1:**
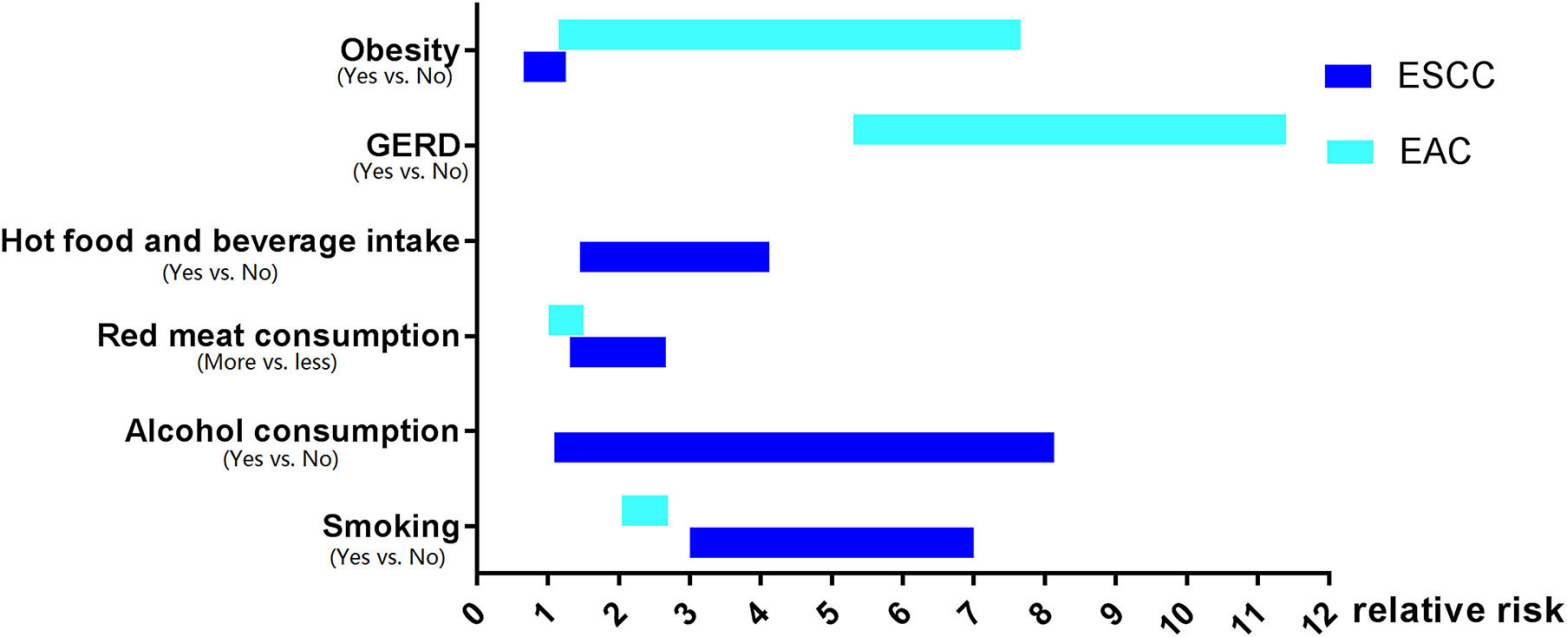
The risk factors profiles for esophageal squamous cell carcinoma and esophageal adenocarcinoma.

The investigation of the risk factors of EC has been carried out for many years. Surprisingly, the risk factors' profiles for ESCC and EAC are quite different even in the same population or the same area (Figure 1).

### Smoking

Tobacco smoking is a risk factor for both ESCC and EAC all around the world, and it was defined as one major cause of EC by the International Agency for Research on Cancer (IARC) (26). Intriguingly, tobacco smoking is more significantly associated with the incidence of ESCC than that of EAC (27). For current smokers, the risk of ESCC increased from three to seven times than those who never smoked (27, 28). Another meta-analysis including 52 studies indicated that the risk of ESCC was dramatically higher in current smokers [risk ratio (RR): 4.18, 95% CI: 3.42–5.12] compared to non-smokers (29). In contrast, smoking cessation could apparently reduce the risk of esophageal carcinogenesis. A reduction in ESCC's risk in ever-smokers was evident after 5 years of termination of smoking when compared to current smokers (RR: 0.59, 95% CI: 0.47–0.75), and this difference was even more significant 10 years (RR: 0.42, 95% CI: 0.34–0.51) and 20 years (RR: 0.34, 95% CI: 0.25–0.47) after smoking cessation (29).

Similarly, tobacco smoking can also elevate the risk of EAC, although the association is not as strong as that of ESCC. Smokers had a nearly 2 fold higher risk of EAC (27, 30). However, the risk of EAC did not show any decrease in ever-smokers after smoking cessation, with a risk ratio of 0.72 (95% CI: 0.52–1.01; follow up for 20 years or longer after smoking cessation) compared to current smokers (29).

### Alcohol Consumption

The effect of alcohol overconsumption on EC carcinogenesis has been widely recognized. In America, over 70% of ESCC patients had alcohol consumption histories (95% CI: 53.3–85.8%) (31). One meta-analysis including 92,000 light drinkers and 60,000 non-drinkers suggested that light drinking increased the risk of ESCC (RR: 1.30; 95% CI: 1.09–1.56), and it was estimated that 24,000 deaths from ESCC were attributed to alcohol drinking globally in 2004 (32). In line with this study, by adjusting age, sex, and tobacco smoking, it was reported that the relative risks of ESCC among light drinkers ( ≤ 12.5 g/day), moderate drinkers (12.5–50 g/day), and heavy drinkers (≥50 g/day) were 1.38 (95% CI: 1.14–1.67), 2.62 (95% CI: 2.07–3.31), and 5.54 (95% CI: 3.92–7.28) compared to non-drinkers, respectively (33).

In China, one study carried out in Huaian, Jiangsu Province, suggested that liquor intake significantly increased the risk of esophageal precancerous lesions [odds ratio (OR): 3.22, 95% CI: 1.28–8.13] (34). Another study suggested that alcohol drinking increased the risk of ESCC by 1.953-fold in the Chinese population (35). Interestingly, Wu et al. reported that consumption of alcohol only increased the risk of ESCC in males but not in females (36), as supported by another study that indicated that only males who drank alcohol had a 2.2-fold (95% CI: 1.79–2.70) higher ESCC risk (37). Mechanistically, a case-control study that enrolled 1,190 patients and 1,883 controls revealed that alcohol consumption could interact with aldehyde dehydrogenase and alcohol dehydrogenase (ADH), resulting in a markedly elevated ESCC incidence (38).

Intriguingly, till now, neither population-based nor hospital-based studies can determine the association between the susceptibility of EAC and alcohol consumption. According to a pool analysis including 1,821 EAC patients and 10,854 controls, alcohol did not increase the risk of EAC even for heavy drinkers who had more than seven drinks per day (14 g of ethanol per drink), whereas this study also indicated that alcohol drinking was significantly associated with an increased risk of ESCC (OR for drinkers more than seven drinks per day: 9.62, 95% CI: 4.26–21.71) (39).

### Dietary

Dietary has been well-defined as the risk factor for EC. Low fruit and vegetable intake accounted for 28.7% (95% CI: 11.1–56.5%) of ESCC and 15.3% (95% CI: 5.8–34.6%) of EAC cases (31). Moreover, increasing diversities of vegetables and fruits could significantly decrease the risk of ESCC, particularly in smokers; however, this phenomenon is not applicable for EAC (40).

Notably, pickled vegetables are also reported to contribute to the carcinogenesis of ESCC, as one study in Jiangsu Province, China, suggested that ingestion of pickled food was correlated with higher incidence of esophageal precancerous lesion (34). Furthermore, another meta-analysis that included 34 studies revealed that consumption of pickled vegetables increased the risk of ESCC ~2 times (41).

Moreover, it was shown that the relative risk of ESCC for people who had more red meat or processed meat was 1.57 (95% CI: 1.26–1.95) or 1.55 (95% CI: 1.22–1.97) compared to people who had less meat consumption (42). This finding was further supported by another pool analysis including seven cohorts and 28 case-control studies, which also indicated that high red meat intake as well as low poultry intake increased the risk of ESCC. Interestingly, high meat consumption, particularly processed meat, was associated with an elevated EAC risk (43).

### Hot Food and Beverage

Numerous studies have identified that hot food and beverage can obviously increase the risk of EC (34, 44–46). A case-control study in northwest China suggested that the OR of ESCC risk among people who preferred hot tea, water, or hot food was 2.23 (95% CI: 1.45–2.90), 2.13 (95% CI: 1.53–2.66), or 2.98 (95% CI: 1.89–4.12), respectively (47). Another study reported that hot beverage, including tea and coffee, increased 2- to 4 fold the risk of EC (44). By adjusting confounding variables, it was demonstrated that intake of hot food and beverage was significantly correlated with a higher risk of ESCC, but not with EAC (46).

### Gastroesophageal Reflux Disease

The prompting effect of gastroesophageal reflux disease (GERD) on EAC carcinogenesis has been very well-characterized. In a medical record-based, case-control study, patients who had a history of GERD had a 2 fold or even higher risk of EAC (47). Another population-based, case-control study suggested the OR of EAC risk among people with recurrent reflux symptom was 7.7 (95% CI: 5.3–11.4) when compared with other people without this symptom (48). Such effect of GERD on EAC carcinogenesis was also observed in a retrospective study in Swedish, as the standardized incidence ratio (SIR) of EAC among GERD patients who did not receive surgery was 6.3 (95% CI: 4.5–8.7) (49). Another meta-analysis including five independent studies revealed that the OR of EAC among patients with weekly or daily GERD symptoms was 4.92 (95% CI: 3.90–6.22) or 7.40 (95% CI: 4.94–11.1), respectively (50). Thus, GERD is a key risk factor of EAC; however, no evidence has been found so far to unveil the correlation between GERD and ESCC carcinogenesis.

### Obesity

Obesity is another confirmed risk factor for EAC. According to the NIH-AARP Diet and Health study, the hazard ratio (HR) of EAC for people with the highest body mass index (BMI) (≥35 kg/m^2^) was 2.11 (95% CI: 1.09–4.09) compared to people with the lowest BMI (<18.5 kg/m^2^) (51). In line with this finding, a pool analysis revealed that the ORs of EAC for people with BMIs of 25–29.9, 30–34.9, 35–39.9, and ≥40 kg/m^2^ were 1.54 (95% CI: 1.26–1.88), 2.39 (95% CI: 1.86–3.06), 2.79 (95% CI: 1.89–4.12), and 4.76 (95% CI: 2.96–7.66), respectively, compared to people with normal BMI (<25 kg/m^2^) (52). Intriguingly, obesity appears to be inversely associated with the risk of ESCC. In a prospective cohort study involving more than 220,000 Chinese, increasing BMI was correlated with decreasing ESCC mortality (53). Consistently, another study in China also suggested that the ESCC HR for people with BMI <18.5, 24–28, and ≥28 kg/m^2^ was 1.21 (95% CI: 1.02–1.43), 0.87 (95% CI: 0.78–0.98), and 0.91 (95% CI: 0.66–1.25), respectively (54).

### Socioeconomic Status

Surprisingly, low socioeconomic status, namely, low income and education, is associated with a higher risk of ESCC. According to a prospective study that enrolled 29,584 individuals in China, participants who received education of 1 to 5 years, completed primary school or middle school education had an RR of ESCC for 0.87 (95% CI: 0.77–0.89), 0.78 (95% CI: 0.64–0.94), or 0.57 (95% CI: 0.45–0.73), respectively, when compared to people without formal education (55). A similar phenomenon was also observed in a case-control study in Iran, as the adjusted ORs of ESCC for people with primary education and high school or beyond were 0.52 (95% CI: 0.27–0.98) and 0.20 (95% CI: 0.06–0.65), respectively, compared to non-educated people (56). Low income is also associated with an elevated ESCC risk. In a Swedish population-based cohort study that enrolled 4,734,227 individuals, participants with a high income had an EC incidence rate ratio (IRR) of 0.74 (95% CI: 0.70–0.79) for men and 0.83 (95% CI: 0.76–0.91) for women when compared to low-income people (57).

Low socioeconomic status was also related to an increased risk of EAC. A case-control study in Swedish suggested that compared to professionals, the risks of EAC were significantly increased in skilled manual workers (OR: 2.4; 95% CI: 1.1–5.3), assistant non-manual employees (OR: 2.3; 95% CI: 1.0–5.3), unskilled manual workers (OR: 3.7; 95% CI: 1.7–7.7), and self-employed (OR: 3.7; 95% CI: 1.7–8.1) (58).

To summarize, the risk factors for ESCC and EAC share very limited similarities. Alcohol consumption, pickled vegetables, hot food, and beverage increase the risk of ESCC but not EAC, whereas GERD increases the risk of EAC only. Although smoking, low intake of fruits and vegetables, high consumption of red, or processed meat, as well as low socioeconomic status increase the risk for both ESCC and EAC, smoking has a much stronger effect on the carcinogenesis of ESCC. Of note, obesity seems to play an opposite role in EAC or ESCC development, which facilitates EAC but negatively correlates with ESCC tumorigenesis.

## Genetics of EC/ESCC

Many studies have investigated the genetic profiles of ESCC and EAC by whole exome sequencing (WES), whole genome sequencing (WGS), chromosomal analysis, and methylation status evaluation. Surprisingly, these studies indicated that there were quite different mutational landscapes between ESCC and EAC (10, 11, 59, 60).

### ESCC

Like many other cancers, TP53 mutations are usually identified in ESCC and play an important role in promoting the development of ESCC (61, 62). Recently, a comprehensive molecular characterization of 164 EC specimens was performed to investigate the molecular signature of ESCC and EAC as well as to improve the classification of EC. The results showed that mutations of TP53, CCND1, SOX2, TP63, PIK3CA, PTEN, NFE2L2, MLL2, ZNF750, NOTCH1, MLL2, FGFR1, and RB1 were significantly enriched in ESCC (11), which were consistent with previous studies (63–65). In this analysis, the 90 ESCCs were classified into three subtypes according to their mutation status, including 50 ESCC1, 36 ESCC2, and 4 ESCC3 (11). Specifically, ESCC1 had a similar genetic characteristic to the classical ESCC, including the alteration in the NRF2 pathway, autophagy pathway, and Hippo pathway. For instance, SOX2 and/or TP63 amplification, YAP1 (11q22.1) amplification, VGLL4/ATG7 deletion, and mutation in NFE2L2 were frequently detected in ESCC1; ESCC2 was defined with more alterations in NOTCH1 and ZNF750, CDK6 amplification, inactivation of KDM6A, KDM2D, PTEN, and PIK3R; ESCC3 harbored more activation in the PI3K pathway and somatic alterations of KMT2D, MLL2, and SMARCA4 (11). Ingenuity pathway assessment suggested that gene mutations in ESCC were mainly involved in cell cycle regulation, Notch, RTK–MAPK–PI3K, and Wnt pathways (66). Another Chinese WES analysis reported that BRCA2 loss-of-function germline mutations were associated with increased ESCC risk (67). Recently, genes involved in chromatin remodeling and cell cycle regulation, such as CDK11A, ARID1A, JMJD6, MAML3, DKN2AIP, and PHLDA1, were also identified with an elevated risk of ESCC (68).

### EAC

The mutation profile of EAC shares limited similarity with ESCC; however, TP53 mutations are also commonly observed in EAC (69, 70). Genomic analysis had revealed that EAC was more similar to gastric cancer in terms of chromosomal instability (71). Unlike ESCC, mutations of ERBB2, VEGFA, EGFR, KRAS, GATA4, SMAD4, CCNE1, GATA6, FGF3/4/19, GATA4/6, CDKN2A, and ARID1A were more frequently recognized in EAC patients (11). Another WES analysis of 149 EAC tumor-normal pairs reported that 26 genes, such as TP53, CDKN2A, SMAD4, ARID1A, PIK3CA, SPG20, TLR4, ELMO1, and DOCK2, were often mutated in EAC, and the activation of the RAC1 pathway contributed to EAC tumorigenesis (72). Meta-analysis of gene expression profiling suggested that EAC could be mainly classified into two subtypes; 24 genes such as SMAD4, SOCS4, and SKAP2 were highly mutated in subtype I EAC, whereas the other 30 genes' mutations, including ARID1A, DCDC1, and IVL, were only detected in subtype II EAC (69).

## Clinical Management of EC

**Figure 2:**
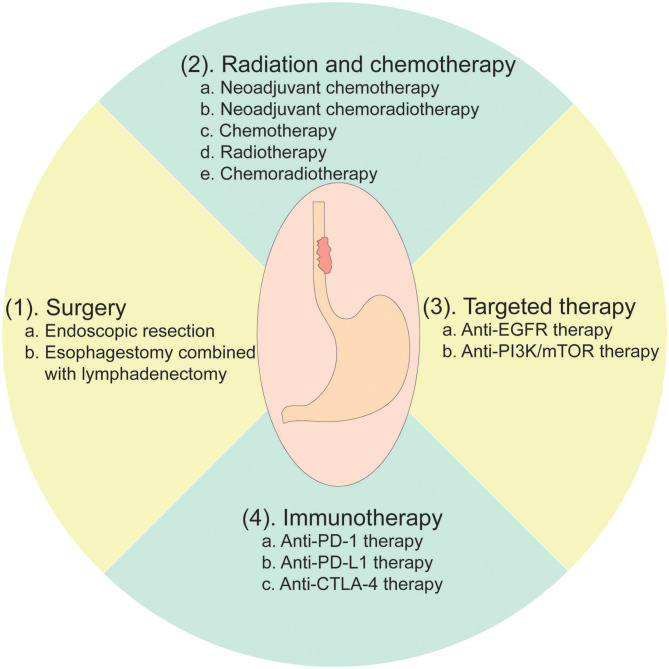
Current treatment options for esophageal cancer.

Currently, the options for EC patients' treatment are very broad. Multimodality treatments, such as endoscopic resection, surgery, chemotherapy (CT), radiotherapy (RT), chemoradiotherapy (CRT), and targeted therapy, are widely applied worldwide (2, 73) (Figure 2).

The treatment strategies are usually determined according to the EC patients' pathological conditions. For early EC limited to the mucosa, endoscopic mucosal resection is the primary treatment option with a 5-year survival rate of 41% (74). After the resection, the specimens should be thoroughly examined for the depth of tumor infiltration, and vascular and nerve invasion (74). For resectable EC with muscle or deeper invasion, esophagectomy combined with lymphadenectomy is the primary treatment strategy, while neoadjuvant CT, RT, or CRT is optional. In a randomized controlled study, patients treated with surgery plus neoadjuvant CT had a median overall survival (OS) of 16.8 months with a 2-year OS rate of 43%, exhibiting a better prognosis compared to those patients who received surgery alone with a median OS of 13.3 months and a 2-year OS rate of 34% (75). Several meta-analyses also suggested that neoadjuvant CT was beneficial to improve OS for EC patients with surgery compared to patients who received surgery alone (76, 77). Although no evidence had shown that neoadjuvant RT could bring survival advantages for patients with resectable EC (78), numerous studies had reported that preoperative CRT definitely improved the survival of patients with EC. For instance, a network meta-analysis including 6,072 EC patients indicated that patients who received neoadjuvant CRT with surgery had better survival compared to those patients who underwent CT with surgery as well as those who received surgery alone (79). This study thus suggested that neoadjuvant therapies combined with surgery are superior treatment strategies compared to surgery followed with adjuvant treatments or surgery alone (79).

Whether targeted therapy has a potential effect on the prognosis of EC is also being wildly investigated (Table 1). However, there are only a few options available for EC patients' targeted therapy, most of which are targeting epidermal growth factor receptor (EGFR) (80), human epidermal growth factor receptor 2 (HER2) (81), or phosphoinositide 3-kinase/mammalian target of rapamycin (PI3K/mTOR) (82). COG, a phase 3 parallel randomized placebo-controlled trial, was aiming to evaluate whether gefitinib (an EGFR tyrosine kinase inhibitor) could be applicable for late-stage EC patients. However, it was shown that gefitinib did not improve the OS of unselected patients with EC (80). Besides, in the RTOG 0436 trial, cetuximab, a specific EGFR monoclonal antibody, was added to concurrent chemoradiation therapy for EC patients who were unable to receive esophagectomy. Unfortunately, the addition of cetuximab to concurrent chemoradiation did not improve clinical complete response and OS in either ESCC or EAC (83). In the ToGa trial, 584 patients with gastric cancer or GEJC were randomly assigned to receive chemotherapy alone or chemotherapy plus trastuzumab, a monoclonal antibody that selectively targeted the extracellular domain of HER2. Patients treated with trastuzumab showed slightly better median OS (13.8 vs. 11.1 months, *p* = 0.0046) (84). Furthermore, in the JACOB trial, 780 patients with metastatic gastric cancer or GEJC were given either pertuzumab (a monoclonal antibody that inhibits HER2) plus trastuzumab in addition to chemotherapy or trastuzumab together with chemotherapy. However, the addition of pertuzumab did not bring any significant survival advantages (85).

**Table 1:** Ongoing clinical trials of targeted therapy for esophageal cancer in China.

**Trail ID**	**Phase**	**Drug**	**Population**	**Primary endpoint**	**Enrollment**	**Center**	**Initiation date**	**Estimated completion date**
**Targeted therapy as salvage treatment**								
NCT02749513	Early Phase 1	Itraconazole	EC	Inhibition of Hedgehog pathway signaling	10	Single	January 2016	December 2021 (estimated primary completion date)
NCT03170310	Phase 2	Apatinib	EC	PFS	60	Single	February 2017	September 2019
NCT03542422	Phase 2	Apatinib	EC	PFS	50	Single	June 2018	May 2019
NCT03770988	Phase 2	Poziotinib	ESCC	ORR	49	Single	April 2019	August 2020
NCT03285906	Phase 2; Phase 3	Apatinib	EC	PFS	30	Single	March 2017	December 2019
NCT03917043	Phase 1	APG-2449	EC	MTD; RP2D	40	Single	May 2019	May 2022
**Targeted therapy combined with chemotherapy/radiotherapy**								
NCT03185988	Phase 2	Trastuzumab + irinotecan	ESCC	RR	100	Single	July 2017	September 2021
NCT01522768	Phase 2	Afatini + paclitaxel	Esophagogastric cancer	ORR; CR; PR	42	Multicenter	March 2012	February 2021
NCT02645864	Phase 1	Apatinib + irinotecan	ESCC	Dose-limiting toxicity; maximum tolerance dose	9	Single	January 2016	December 2017
NCT01463605	Phase 2	Nimotuzumab + radiotherapy	EC	Safety	30	Single	October 2011	October 2014
**Targeted therapy combined with chemoradiotherapy**								
NCT01034189	Phase 3	Cetuximab + paclitaxel/cisplatin + radiotherapy	EC	cRR	62	Single	October 2008	June 2012
NCT04207918	Phase 2	Nimotuzumab + S-1 + radiotherapy	EC	Local control rate	58	Single	November 2019	August 2022
NCT02409186	Phase 3	Nimotuzumab + chemoradiotherapy vs. placebo + chemoradiotherapy	ESCC	OS	200	Multicenter	March 2015	December 2021
**Targeted therapy combined with immunotherapy**								
NCT03736863	Phase 2	Apatinib + SHR-1210	EC	ORR	45	Single	April 2019	April 2021
**Targeted therapy combined with immunotherapy and chemotherapy**								
NCT03615326	Phase 3	Pembrolizumab + trastuzumab + chemotherapy vs. placebo + trastuzumab + chemotherapy	GEJC	PFS; OS	732	Multicenter	October 2018	March 2024
NCT03603756	Phase 2	SHR-1210 + apatinib + irinotecan vs. SHR-1210 + apatinib + paclitaxel + nedaplatin	ESCC	PFS	30	Single	July 2018	March 2021

*EC, esophageal cancer; ESCC, esophageal squamous cell carcinoma; GEJC, gastroesophageal junction carcinomas; PFS, progression-free survival; ORR, objective response rates; MTD, maximum tolerated dose; RP2D, recommended phase 2 dose; RR, response rate; CR, complete response; PR, partial response; cRR, clinical response rate; OS, overall survival*.

Nevertheless, surgery, CT, RT, CRT, or targeted therapy can only bring mild survival advantages to EC patients, making this disease one of the leading causes for cancer-related death.

## Immunotherapy for EC/ESCC

Recently, immunotherapy has opened a new era for cancer treatment with extraordinary therapeutic benefits in certain cancer patients. Clinically, there are two major immunotherapy options for EC patients, which are anti-programmed cell death 1 ligand 1 (anti-PD-L1)/anti-programmed cell death 1 (anti-PD-1) and anticytotoxic T-lymphocyte-associated antigen-4 (anti-CTLA-4) therapy. A comprehensive overview for those ongoing clinical trials of immunotherapy for Chinese EC patients are listed in Table 2.

**Table 2:** Ongoing clinical trials of immunotherapy for esophageal cancer in China.

**Trail ID**	**Phase**	**Design**	**Population**	**Primary endpoint**	**Enrollment**	**Center**	**Initiation date**	**Estimated completion date**
**Immunotherapy as salvage treatment**								
NCT03811379	Phase 2	Toripalimab	Small Cell Carcinoma of Esophagus	ORR	43	Single	November 2018	December 2021
NCT03019588	Phase 3	Pembrolizumab vs. paclitaxel	GEJC	OS; PFS	360	Multicenter	February 2017	June 2021
NCT03941626	Phase 1; Phase 2	CAR-T/TCR-T	EC	Safety	50	Single	September 2019	December 2020
NCT03638206	Phase 1; Phase 2	CAR-T/TCR-T	EC	Safety	73	Single	March 2018	March 2023
NCT03013712	Phase 1; Phase 2	EpCAM targeted CAR-T	EC	Toxicity	60	Single	January 2017	December 2020
NCT02693236	Phase 1; Phase 2	DC vaccine + CIK cells	EC	ORR	30	Single	August 2014	November 2016
NCT02743494	Phase 3	Nivolumab vs. placebo	EC; GEJC	DFS	760	Multicenter	May 2016	October 2025
NCT03706326	Phase 1;Phase 2	anti-MUC1 CAR T alone vs. anti-MUC1 CAR T + PD-1 knockout engineered T cells vs. PD-1 knockout engineered T cell only	EC	Safety and tolerability	20	Single	September 2018	September 2021
NCT02662348	Phase 1	recombinant Human interleukin-2 + HER2Bi-armed T-cell transfusion	EC	Safety and toxicities	6	Single	February 2016	November 2017
NCT02457650	Phase 1	Anti-NY ESO-1 TCR-T	EC	Safety and toxicities	36	Single	April 2015	December 2019
NCT04074447	Observational	The efficacy of immunodetection point inhibitors for advanced EC	EC	The proportion of ctDNA content decreased in patients with good therapeutic effect	80	Single	May 2019	May 2021
**Immunotherapy combined with neoadjuvant chemotherapy/chemotherapy**								
NCT03985670	Phase 2	Neoadjuvant chemotherapy (paclitaxel + cisplatin) and teripalimab in the same day vs. neoadjuvant chemotherapy (paclitaxel + cisplatin) followed by teripalimab	ESCC	pCRR	30	Single	July 2019	April 2023
NCT02644863	Phase 2	DC-CIK + paclitaxel + cisplatin vs. paclitaxel + cisplatin	EC	OS	60	Single	December 2015	May 2019
NCT03946969	Phase 1; Phase 2	Sintilimab + paclitaxel + cisplatinum + S-1	EC	AE	40	Single	May 2019	October 2022
NCT04225364	Phase 2	Camrelizumab + neoadjuvant chemotherapy (paclitaxel + cisplatin)	ESCC	PCR	50	single	January 2020	June 2023
NCT03615326	Phase 3	Pembrolizumab + trastuzumab + chemotherapy vs. placebo + trastuzumab + chemotherapy	GEJC	PFS; OS	732	Multicenter	October 2018	March 2024
NCT03691090	Phase 3	SHR-1210 + paclitaxel + cisplatin vs. placebo + paclitaxel + cisplatin	EC	PFS; OS	548	Single	December 2018	October 2021
**Immunotherapy combined with neoadjuvant radiotherapy/radiotherapy**								
NCT01691664	Not applicable	Radiotherapy + DC-CIK cellular therapy vs. radiotherapy only	EC	DFS	40	Single	September 2012	June 2022
NCT03011255	Phase 2	Radiotherapy + peptide-specific CTL	EC	Local control	20	Single	December 2016	December 2019
NCT03200691	Phase 2	SHR-1210 + neoadjuvant radiotherapy	ESCC	pCRR	21	Single	August 2017	July 2020
NCT03187314	Phase 2	SHR-1210 + radiation	EC	Local control	21	Single	June 2017	December 2019
**Immunotherapy combined with neoadjuvant chemoradiotherapy/chemoradiotherapy**								
NCT01691625	Not applicable	Chemoradiation only vs. chemoradiotherapy + DC-CIK immunotherapy	EC	QOL	50	Single	September 2012	December 2021
NCT04005170	Phase 2	Toripalimab + radiothetapy + paclitaxel/cisplatin	ESCC	cCCR	42	Single	June 2019	December 2022
NCT03671265	Not applicable	SHR-1210 + chemotherapy + radiotherapy	ESCC	AE	20	Single	September 2018	September 2021
NCT04177875	Phase 2	Teripalimab + chemoradiation	EC	MPR; ORR	44	Single	May 2019	April 2022
NCT03940001	Early Phase 1	Sintilimab + neoadjuvant chemoradiotherapy	ESCC	toxicity; pCRR; MPR	20	Single	May 2019	May 2022
NCT04006041	Phase 2	Toripalimab + neoadjuvant chemoradiotherapy	ESCC	pCCR	44	Single	June 2019	December 2020
NCT04177875	Phase 2	Teripalimab + chemoradiation	EC	MPR; ORR	44	Single	May 2019	April 2022
NCT04084158	Phase 2	Chemoradiation vs. triprizumab + chemoradiation	ESCC	PFS	100	Single	September 2019	December 2021
**Immunotherapy combined with targeted therapy**								
NCT03736863	Phase 2	Apatinib + SHR-1210	EC	ORR	45	Single	April 2019	April 2021
**Immunotherapy combined with targeted therapy and chemotherapy**								
NCT03603756	Phase 2	SHR-1210 + apatinib + irinotecan vs. SHR-1210 + apatinib + paclitaxel + nedaplatin	ESCC	PFS	30	Single	July 2018	March 2021

*GEJC, gastroesophageal junction carcinomas; EC, esophageal cancer; ESCC, esophageal squamous cell carcinoma; CAR-T, chimeric antigen receptor T-cell immunotherapy; TCR-T, T-cell receptor-engineered T-cell immunotherapy; DC, dendritic cells; CIK, cytokine-induced killer cells; PD-1, programmed cell death 1; ORR, objective response rates; OS, overall survival; PFS, progression-free survival; DFS, disease-free survival; pCRR, pathological complete response rate; QOL, the quality of life; cCCR, clinical complete response rate; AE, adverse events; MPR, major pathological response*.

### Anti-PD-L1 or Anti-PD-1 Therapy

PD-L1, a molecule that locates on the tumor cells' surface, can bind to PD-1, which is expressed on the T cells' membrane, resulting in inhibition of T-cell function thus, contributing to tumor cell escape from immunosurveillance (86).

Pembrolizumab is a PD-1 inhibitor, which was first approved by FDA for treating patients with advanced or unresectable melanoma (87). KEYNOTE-028, a multicohort phase IB study, was designed to investigate the potential therapeutic effect of pembrolizumab on patients with PD-L1-positive advanced solid tumors. In the EC cohort, patients were treated with pembrolizumab every 2 weeks for up to 2 years or until confirmed disease progression or intolerable toxicity. The overall response rate was 30% (95% CI: 13–53%), and the median duration of response was 15 months (range from 6 to 26 months) (88). KEYNOTE-180, a phase 2, open-label, interventional, and single-arm study, was designed to evaluate the efficacy and safety of pembrolizumab for advanced, metastatic ESCC, EAC, or gastroesophageal junction adenocarcinoma patients with disease progression after two or more lines of systematic therapies. The objective response rate was 9.9% (95% CI: 5.2–16.7%) among all patients (12 of 121), while the median duration of response was not achieved (range, 1.9–14.4 months). In detail, the objective response rate was 14.3% (95% CI: 6.7–25.4%) for ESCC patients (9 of 63), 5.2% (95% CI: 1.1–14.4%) for EAC patients (3 of 58), 13.8% (95% CI: 6.1–25.4%) for all patients with PD-L1-positive tumors (8 of 58), and 6.3% (95% CI: 1.8–15.5%) for all patients with PD-L1-negative tumors (4 of 63) (89), suggesting a quite low response rate of EC patients to anti PD-1/PD-L1 therapy.

### Anti-CTLA-4 Therapy

CTLA-4 is a transmembrane receptor on T cells, which inactivates early stages of T cells by interacting with CD80 or CD86 (86). Currently, the immunotherapy targeting CTLA-4 has been wildly used for treating various cancer patients, including EC (90, 91). The efficacy of tremelimumab, a monoclonal antibody against CTLA-4, was previously investigated for treating metastatic gastric cancer and EAC patients; unfortunately, a phase II clinical trial that enrolled 18 such patients treated with tremelimumab revealed no effects on progression-free survival (PFS) or OS (92). In the CheckMate-032 study, a multicohort, phase I/II trial, 160 patients with locally advanced or metastatic chemotherapy-resistant EC, gastric, or gastroesophageal junction cancer were treated with either (i) nivolumab (3 mg/kg), (ii) nivolumab (1 mg/kg) plus ipilimumab (3 mg/kg), or (iii) nivolumab (3 mg/kg) plus ipilimumab (1 mg/kg). The objective response rates were 12% (95% CI: 5–23%), 24% (95% CI: 13–39%), and 8% (95% CI: 2–9%), the 12-month PFS rates were 8, 17, and 10%, and the 12-month OS rates were 39, 35, and 24% for the abovementioned three groups, respectively. This study demonstrated for the first time that combined anti-PD-L1/PD1 and anti-CTLA-4 therapy could provide clinical benefits and durable antitumor activity for advanced or metastatic chemotherapy-resistant EC, gastric, or gastroesophageal junction cancer patients (93); however, more randomized controlled trials were required to validate the efficacy and safety of anti-CTLA-4 therapy for EC patients.

## Conclusions

Although the incidence of EC is decreasing in the last few decades, it remains as one of the leading causes of cancer-related deaths in China. ESCC and EAC, two subtypes of EC, share very limited similarity in risk factors as well as genetic mutation profile, suggesting that they are actually two distinct diseases; thus, the treatment strategy and prognosis for these two EC subtypes could be quite different. Until now, the most efficient strategy to treat EC patients is combining esophagectomy and lymphadenectomy; therefore, early screening and diagnosis for EC patients are of utmost importance. Currently, the majority of EC patients are diagnosed at a late stage with local or distant metastasis, and many available therapies, including targeted therapy and immunotherapy, do not bring satisfying survival advantages for these patients as for other cancer populations. Combining different therapies together represents a promising strategy in the future for late-stage EC patients, although extensive clinical trials are demanded in a randomized, multi-center fashion. The comprehensive understanding of EC tumorigenesis is still lacking due to limited research systems, as most findings of EC development are generated from *in vitro* cultured EC cell lines. We, therefore, advocate newly emerged tools, such as EC patient-derived organoid (EC-PDO) (94, 95) and spontaneous EC animal models (96) to seek ultimate personalized therapy for EC patients.

## Author Contributions

JY and XL collected the data of clinical trials and drafted the manuscript. SC and XD coordinated and edited the drafting of the manuscript. KC and SR revised and edited the final version of the manuscript. All authors read and approved the final manuscript.

### Conflict of Interest

The authors declare that the research was conducted in the absence of any commercial or financial relationships that could be construed as a potential conflict of interest.

## Abbreviations

EC: esophageal cancer
ESCC: esophageal squamous cell carcinoma
EAC: esophageal adenocarcinoma
GEJC: gastroesophageal junction carcinomas
GC: gastric cancer
NCCRC: National Central Cancer Registry of China
IARC: the International Agency for Research on Cancer
RR: risk ratio
CI: confidence interval
OR: odds ratio
ADH: alcohol dehydrogenase
GERD: gastroesophageal reflux disease
SIR: standardized incidence ratio
IRR: incidence rate ratio
BMI: body mass index
WES: whole exome sequencing
WGS: whole genome sequencing
CT: chemotherapy
RT: radiotherapy
CRT: chemoradiotherapy
OS: overall survival
EGFR: epidermal growth factor receptor
hEGFR: human epidermal growth factor receptor
PI3K/mTOR: phosphoinositide 3-kinase/mammalian target of rapamycin
anti-PD-L1: anti-programmed cell death 1 ligand 1
anti-PD-1: anti-programmed cell death 1
anti-CTLA4: anticytotoxic T lymphocyte-associated antigen-4
PFS: progression-free survival
PDO: patient-derived organoid.
